# *In silico* predicted structural and functional insights of all missense mutations on 2B domain of *K1/K10* causing genodermatoses

**DOI:** 10.18632/oncotarget.10599

**Published:** 2016-07-13

**Authors:** Santasree Banerjee, Qian Wu, Yuyi Ying, Yanni Li, Matsuyuki Shirota, Dante Neculai, Chen Li

**Affiliations:** ^1^ Department of Cell Biology and Medical Genetics, School of Medicine, Zhejiang University, Hangzhou, China; ^2^ BGI-Shenzhen, Shenzhen, China; ^3^ Department of Applied Information Sciences, Graduate School of Information Sciences, Tohoku University, Sendai, Japan

**Keywords:** genodermatoses, missense mutations, coiled-coil heterodimer, in silico analysis, Pathology Section

## Abstract

The *K1* and *K10* associated genodermatoses are characterized by clinical symptoms of mild to severe redness, blistering and hypertrophy of the skin. In this paper, we set out to computationally investigate the structural and functional effects of missense mutations on the 2B domain of *K1/K10* heterodimer and its consequences in disease phenotype. We modeled the structure of the *K1/K10* heterodimer based on crystal structures for the human homolog *K5/K14* heterodimer, and identified that the missense mutations exert their effects on stability and assembly competence of the heterodimer by altering physico-chemical properties, interatomic interactions, and inter-residue atomic contacts. Comparative structural analysis between all the missense mutations and SNPs showed that the location and physico-chemical properties of the substituted amino acid are significantly correlated with phenotypic variations. In particular, we find evidence that a particular SNP (*K10*, p.E443K) is a pathogenic nsSNP which disrupts formation of the hydrophobic core and destabilizes the heterodimer through the loss of interatomic interactions. Our study is the first comprehensive report analyzing the mutations located on 2B domain of *K1/K10* heterodimeric coiled-coil complex.

## INTRODUCTION

Genodermatoses are inherited skin disorders primarily caused by mutation of keratin genes. Mutations in *K1/K10* cause a large group of clinically and genetically heterogeneous genodermatoses characterized by keratinocyte fragility, blistering, and thickening of the palmoplantar epidermis [1].

Keratins are a family of structurally related heterodimers, which act as cytoskeletal scaffolding in all epithelial cells. Keratins can form a heteropolymer of either type I (acidic, *K9-K20*, 17q11-q21) or type II (neutral-basic, *K1-K8*, 12q11-q13) which are expressed in a tissue and differentiation-specific pattern. *K1/K10* is expressed in the suprabasal cells of stratified and cornified epithelia, including palms and soles. Three hundred and fifty unique keratin mutations across 21 keratin genes, leading to 40 types of genodermatoses have been reported [2].

Only a few *in silico* structural studies have been performed for keratin genes associated genodermatoses. Jeřábková *et al.* have identified mutations in both *K5* and *K14* genes associated with Epidermolysis bullosa simplex (EBS) affected families in the Czech Republic [3]. Using the 3D structure of vimentin (PDB ID: 1GK4), they considered the structural effects of nine mutations (p.L463P, p.I467L, p.I467T, p.T469P on the 2B domain of *K5* and p.L408M, p.E411del, p.A413T, p.Y415C, p.Y415H on the one of *K14*) using molecular dynamics. We have previously extended this research through a comprehensive structural analysis of missense mutations on the 2B domain of *K5/K14* using the recent identification of the *K5/K14* coiled-coil crystal structure in 2012 [4]. Recently, Mirza *et al.* has provided great structural and functional insights into the understanding of two mutations (p.Q434del and p.R441P) of *K10* [5].

In this paper, we combine a comprehensive *in silico* analysis with relevant structural studies in order to better understand the phenotypic effects of missense mutations on the protein structure. The main contributions are collecting all reported missense mutations (on the 2B domain of *K1* and *K10*) from public databases and existing literature, as well as investigating the correlation between phenotype and protein structure from physico-chemical and structural viewpoints. We characterize the structural changes which affect the stability of the keratin coiled-coil heterodimer complex and eventually lead to the phenotypic variations.

The paper is organized as follows: in the Materials and Methods section we briefly introduce *K1/K10* associated genodermatoses and its phenotype according to varying severities, and describe the methods used to build the homology model of *K1/K10* coiled-coil complex through the *K5/K14* crystal structure. We analyze all missense mutations (21 missense mutations in total, *K1*: 11, *K10*: 10) on the 2B domain after data integration. In the Results section, we investigate the characteristics of all 21 missense mutations with respect to (i) position/location of the mutations, (ii) disease severity and (iii) disease diversity. We characterize and categorize four specific groups and then undertake an *in silico* analysis in detail to reveal the correlation between the phenotype and structural effects in an integrated way. In the Discussion section, we discuss other possible significant epigenetic factors and modifications that affect disease severity and address their contributions.

## RESULTS

### Homology modeling

**Figure 1:**
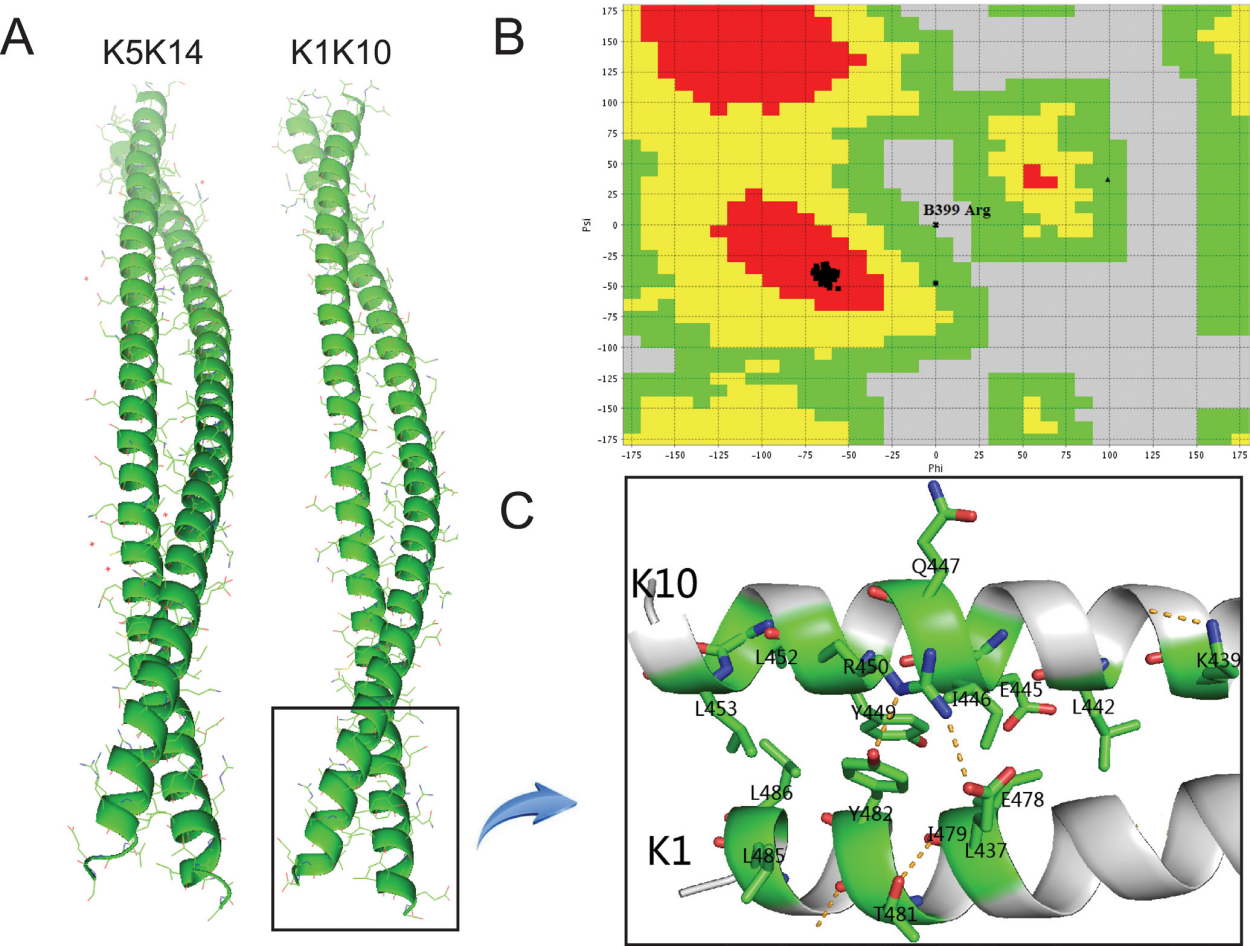
A *K1/K10* heterodimer modeled by MODELLER **B.** Ramachandran plot of *K1/K10* predicted model. It can be seen that only one ‘Arg’ residue is present in the disallowed region of the plot and most of the residues are present in the allowed region and two of them are generously allowed. **C.** Schematic representation of 2B domain for *K1/K10* heterodimer with missense mutations.

The homology modeling of *K1/K10* coiled-coil structure was conducted with MODELLER. The α-helix structure of the resulting *K1/K10* heterodimer is structurally quite similar with the *K5/K14* heterodimer (Figure 1A). The structural quality of the *K1/K10* homology model was validated with Ramachandran plots and RMSD of the alignments using PROCHECK and VADAR. Figure 1B shows that our built model has high quality according to the resulting Ramachandran plots which contain ≥ 90% of their residues in allowed regions. The RMSD calculated by PyMOL is 1.646 Å, which is much lower than general cut-off value, 2.8 Å.

### *In silico* study

**Figure 2:**
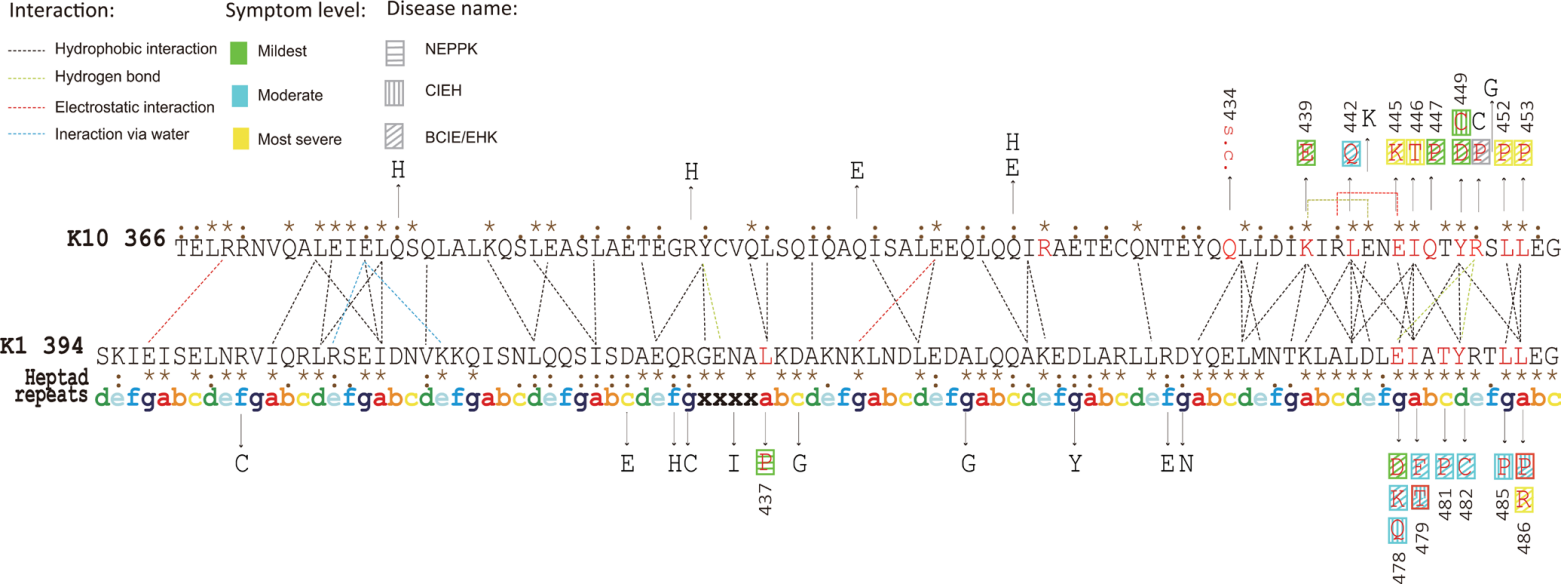
Structural representation and interactions of the heterodimeric complex The symbols in this figure are used as follows: (i) point mutations derived from published sources are denoted by black arrows; (ii) the point mutations causing relevant disease are shown in red letters and severities of phenotypes are depicted by three different colors: green, blue and yellow; each different disease is represented by three different patterns; (iii) amino acid substitutions causing two phenotypes are denoted with two patterns simultaneously surrounded with a red frame; (iv) the SNPs are shown in black letters; (v) heptad repeats of ‘abcdefg’ stand for a cycle of the a-helix and ‘xxxx’ denote the stutter region; (vi) consensus sequence from multiple alignment is given beside the amino acid sequence, hierarchically, ‘.’ is conserved, ‘:’ is more conserved and ‘*’ denotes the most evolutionarily conserved; (vii) the background color in grey denotes unknown symptoms level.

We analyzed the effects of all 21 missense mutations (*K1*: 11, *K10*: 10) on the 2B domain of the *K1/K10* coiled-coil complex as shown in Figure 2 and Table 1. The results clearly suggest that the missense mutations on the 2B domain of *K1/K10* cause genodermatoses by disrupting the inter-atomic hydrogen bond, hydrophobic interactions, and interatomic steric clashes. We categorize these twenty-one missense mutations into four groups.

*K1 K10*, *e.g.* p.E478K and p.E478Q of *K1* causes BCIE/EHK and CIEH respectively; p.Y449D and p.Y449C of *K10* causes BCIE/EHK and CIEH respectively.

This is the most common feature of several pathogenic missense mutations. In this case, the structure and physico-chemical characteristics of the substituted amino acid are the most significant limiting factor for resulting phenotype. Our analyses focused on three criteria, *i.e.* physico-chemical properties, interatomic interaction, inter-residue atomic contacts. It has been reported that two pathogenic variants, *i.e.* p.E478K and p.E478Q of *K1* cause severe BCIE/EHK and severe CIEH respectively while substitution of this residue by aspartic acid p.E478D causes a mild phenotype of BCIE/EHK. This is because the p.E478 residue is located at position “g” of the heptad repeat and evolutionarily highly conserved. Therefore a mutation at this site is expected to be highly disruptive to filament formation and cause severe clinical symptoms. As an example, we will discuss the correlation between phenotype and structural effects of p.E478 mutations in detail.

**Table:** The change of physico-chemical properties of substituted amino acid which causes subsequent alterations in atomic interaction according to computational analyses

cDNA	AA	#1	Characteristics change		Change of interactions	
			Wild type	Mutated AA	Hydrophobic interaction	H-bond
c.1310T>C	K1-p.L437P	NEPPK	Aliphatic, Hydrophobic, Neutral	Hydrophobic, Neutral	Loss of hydrophobic interaction with K10 Q403 and L404	-
c.1432G>A	K1-p.E478K	BCIE/EHK	Polar, Hydrophilic, Charged(-)	Polar, Hydrophilic, Charged(+)	-	Hydrogen bond is broken with K10 R450
c.1432G>C	K1-p.E478Q	CIEH	Polar, Hydrophilic, Charged(-)	Polar, Hydrophilic, Neutral	-	Hydrogen bond is broken with K10 R450
c.1434G>T	K1-p.E478D	BCIE/EHK	Polar, Hydrophilic, Charged(-)	Polar, Hydrophilic, Charged(-)	-	Hydrogen bond is broken with K10 R450
c.1435A>T	K1-p.I479F	BCIE/EHK	Aliphatic, Hydrophobic, Neutral	Aromatic, Hydrophobic, Neutral	Loss of hydrophobic interaction with K10 L442, E445 and I446	-
c.1436T>C	K1-p.I479T	BCIE/EHK/CIEH	Aliphatic, Hydrophobic, Neutral	Polar, Hydrophilic, Neutral	Loss of hydrophobic interaction with K10 L442, E445 and I446	-
c.1441A>C	K1-p.T481P	BCIE/EHK	Polar, Hydrophilic, Neutral	Hydrophobic, Neutral	-	-
c.1445A>G	K1-p.Y482C	BCIE/EHK	Aliphatic, Hydrophobic, Polar	*Polar*, Hydrophilic, Neutral	Loss of hydrophobic interaction with K10 I446 and R450	Hydrogen bond is broken with K10 R450
c.1454T>C	K1-p.L485P	CIEH	Aliphatic, Hydrophobic, Neutral	Hydrophobic, Neutral	Loss of hydrophobic interaction with K10 L453	-
c.1457T>C	K1-p.L486P	BCIE/EHK/CIEH	Aliphatic, Hydrophobic, Neutral	Hydrophobic, Neutral	Loss of hydrophobic interaction with K10Y449 and L453	-
c.1457T>G	K1-p.L486R	BCIE/EHK	Aliphatic, Hydrophobic, Neutral	Polar, Hydrophilic,Charged(+)	Loss of hydrophobic interaction with K10Y449 and L453	-
c.1315A>G	K10-p.K439E	BCIE/EHK	Polar, Hydrophilic, Charged(+)	Polar, Hydrophilic, Charged(-)	Loss of hydrophobic interaction with K1 L468, T471 and L475	Hydrogen bond is broken with K10 E443
c.1325T>A	K10-p.L442Q	BCIE/EHK	Aliphatic, Hydrophobic, Neutral	Polar, Hydrophilic, Neutral	Loss of hydrophobic interaction with K1K472, L475, D476 and I479	-
c.1333G>A	K10-p.E445K	BCIE/EHK	Polar, Hydrophilic, Charged(-)	Polar, Hydrophilic, Charged(+)	Loss of hydrophobic interaction with K1 I479	-
c.1337T>C	K10-p.I446T	CIEH	Aliphatic, Hydrophobic, Neutral	Polar, Hydrophilic, Neutral	Loss of hydrophobic interaction with K1E478, I479, Y482	-
c.1340A>C	K10-p.Q447P	BCIE/EHK	Polar, Hydrophilic, Neutral	Hydrophobic, Neutral	-	-
c.1345T>G	K10-p.Y449D	BCIE/EHK	Aliphatic, Hydrophobic, Polar	Polar, Hydrophilic, Charged(-)	Loss of hydrophobic interaction with K1 L486	-
c.1346A>G	K10-p.Y449C	CIEH	Aliphatic, Hydrophobic,Polar	Polar, Hydrophilic, Neutral	-	-
c.1349G>C	K10-p.R450P	BCIE/EHK	Polar,Hydrophilic,Charged(+)	Hydrophobic, Neutral	-	Hydrogen bonds are broken with K1E478 and Y482
c.1355T>C	K10-p.L452P	BCIE/EHK	Aliphatic, Hydrophobic, Neutral	Hydrophobic, Neutral	Loss of hydrophobic interaction with K1 L486	-
c.1358T>C	K10-p.L453P	BCIE/EHK	Aliphatic, Hydrophobic, Neutral	Hydrophobic, Neutral	Loss of hydrophobic interaction with K1 L486	-

#1 disease name

**Figure 3:**
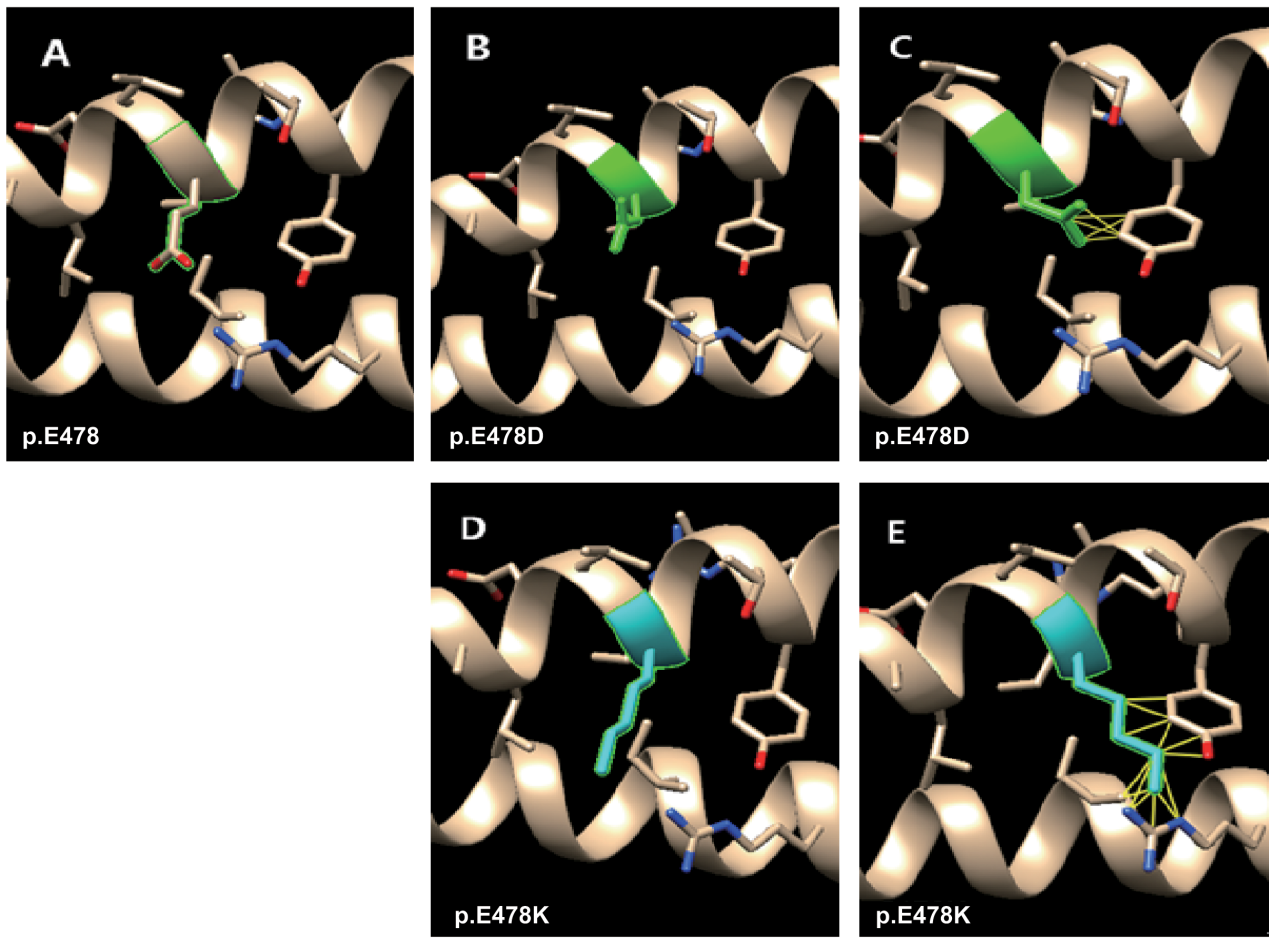
Molecular dynamics snapshot showing the effects of the point mutations (p.E478D and p.E478K of *K1*) on the *K1/K10* coiled-coil complex Mutations in green and blue denote mild and moderate phenotypes respectively. **A.** wild type of p.E478; **B.** the first rotamer of p.E478D (no clash); **C.** the third rotamer of p.E478D (5 clashes); **D.** The first rotamer of p.E478K (no clash); **E.** the second rotamer of p.E478K (13 clahses).

Structural analyses revealed that the wild type amino acid p.E478 is polar, hydrophilic and negatively charged; interacts with p.R450 of *K10* through a hydrogen bond (refer to Table 1); and lacks interatomic clashes (Figure 3A). Mutation p.E478D leads to the loss of hydrogen bond with p.R450 of *K10*. However, p.E478D has nine rotamers and the first one (60.9% probability) has no clash (Figure 3B and Suppl. Table 1), whereas the third one (12.7% probability) has five clashes (Figure 3C) with p.Y482 of *K1* before the energy minimization. The structural effect of p.E478D replaces the Glutamic acid (E) at position 478 has mutated to aspartic acid (D) which shortens of the side chain by only one carbon atom and is therefore a conservative alteration. *In vitro* keratin filament assembly experiments demonstrated that residual filament-forming ability was present in the p.E478D mutant peptide [6]. This residual filament-forming ability is linked to the mild phenotype of p.E478D with superficial blisters resembling superficial EI (epidermal ichthyosis/ichthyosisbullosa of Siemens) [7].

On the other hand, for p.E478K, the hydrogen bond becomes broken with p.R450 of *K10* as Glutamic acid (E) is negatively charged and Lysine (K) is positively charged. In addition, Lysine has more than 20 rotamers (Suppl. Table 1), and the first one (22.1% probability) has no clash (Figure 3D), whereas the second one (19.7% probability) has 13 clashes (Figure 3E) with p.Y482 of *K1* and p.I446, p.R450 of *K10* before the energy minimization. This is probably the reason that p.E478K causes a severe phenotype compared to p.E478D.

*e.g.* p.I479T of *K1* causes BCIE/EHK, CIEH and EPPK respectively in different populations.

This is the most striking feature for missense mutations located on the 2B domain of *K1*. p.I479T of *K1* causes BCIE/EHK, CIEH respectively in different populations. It has been reported that a pathogenic variant p.I479T of *K1* causes severe BCIE/EHK in USA [8, 9] and China [10] while the same variant causes severe CIEH in Germany [7], and finally it was reported to cause a moderate form of EPPK in UK [11]. Thus genotype-phenotype correlation showed that the phenotypic effects of p.I479T variant are population specific.

p.I479 is evolutionarily highly conserved across type I and type II keratins as well as other intermediate filaments, which strongly suggests that any minor changes at this amino acid position will lead to severe conformational alterations in the *K1* protein, disrupting formation of the *K1/K10* coiled-coil complex. Moreover, p.I479 residue is located at position “a” of the heptad repeat which is occupied by a polar, hydrophobic amino acid. These residues are thought to interact with amino acids located in the “d” position of the partner molecule through a hydrophobic interaction that can stabilize the heterodimeric coiled-coil structure. The non-conservative substitution of isoleucine by threonine, which is a polar amino acid, does not cause any change in charge, but alters the length of the side chain. Although this isoleucine residue is located two amino acids upstream of the highly conserved *TYRRLLEGEE* termination motif, it could still affect the conformation of the coiled-coil in this critical region of the heterodimer [9].

As we know, the mutated amino acid possesses different rotamers and the number of rotamers is directly correlated with the length of the side chain in the substituted amino acid. Each rotamer has a specific number of inter-atomic clashes which can be considered population specific: *i.e.*, individuals from different populations have different rotamers in their keratinocytes. Furthermore, there may be some other underlying environmental, epigenetic and stochastic factors which cause these geographical or spatial variations in terms of phenotypes. The population-specific inter-individual phenotypic diversity in the severity of the disease symptoms might be caused by the differences in the genetic background and the presence of “modifying genes” in each of the individuals.

*K1* *e.g.* both p.E478K/D and p.L486P/R of *K1* causes BCIE/EHK.

This is also a significant feature for missense mutation of both *K1* and *K10*, *i.e.* the different mutations cause BCIE/EHK with a similar phenotypic range. Two missense mutations (p.L486P and p.L486R of *K1*) have been reported to both cause BCIE/EHK. The “L” residue at position 486 of the *K1* polypeptide chain is evolutionarily well conserved across all type II keratin proteins (see Figure 2). Two missense mutations have been reported at this position (p.L486P and p.L486R) with the same phenotype. The wild type “L” is aliphatic, hydrophobic and neutral. It has two hydrophobic interactions with p.Y449 and p.L453 of *K10*. Both the substituted amino acids (“P” and “R”) have the same physico-chemical properties, *i.e.* a polar, hydrophilic and neutral. Due to the lack of hydrophobicity, both missense mutations result in loss of two hydrophobic interactions with *K10* (Table 1).

**Figure 4:**
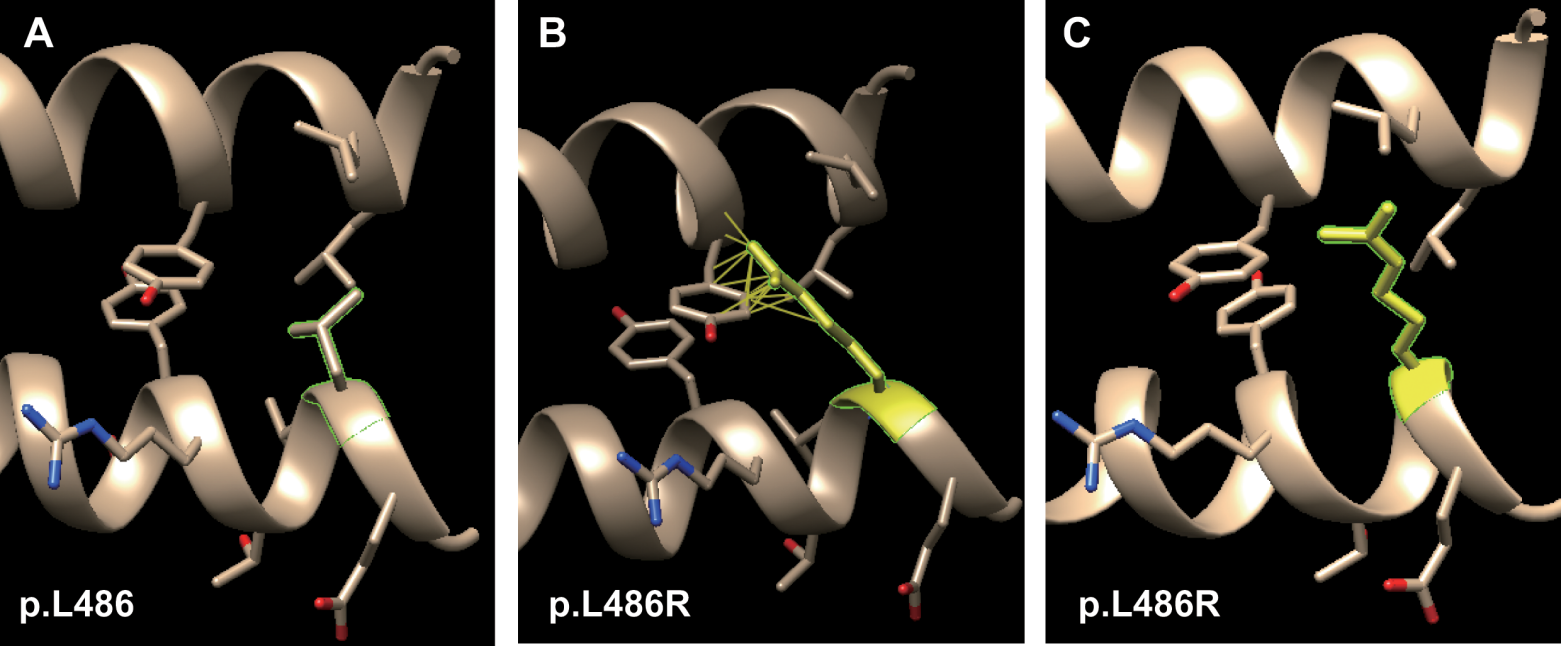
Molecular dynamics snapshot of the effects for point mutations (p.L486R of *K1*) on the *K1/K10* coiled-coil heterodimer complex **A.** wild type of p.L486; **B.** the first rotamer of p.L486R (15 clashes); **C.** the third rotamer of p.L486R (no clash).

The result of inter-residue atomic contact analysis showed that “R” has more than 20 rotamers and the first one (14.2% probability) has 15 clashes with p.Y449 of *K10* (Figure 4B), whereas the third one (5.9% probability) has no clash (Figure 4C). All these above-mentioned reasons, *i.e.*, same physico-chemical properties, loss of hydrophobic interaction and interatomic steric clashes, explain how these two different substituted amino acids in a same position results in the same phenotype.

*K1/K10*which is the most common features of disease causing mutations.

### SNPs: Are they disease causing?

A single-nucleotide polymorphism is a single nucleotide DNA sequence variation with high frequency in normal population. There are two types of SNPs in coding regions: synonymous and nonsynonymous. Here, the wild type amino acids of these five SNPs (p.R403C, p.D457Y, p.D464N of *K1* and p.R399H, p.E443K of *K10*) are evolutionarily highly conserved among all 19 studied SNPs (*K1*: 11, *K10*: 8) on the 2B domain of *K1/K10*. Their wild-type amino acids have inter-chain interactions and therefore even subtle changes can destabilize the *K1/K10* monomer and prevent the formation of *K1/K10* heterodimer.

Interestingly, we identified the SNP p.E443K in the *K10* polypeptide chain as the most damaging SNP and a pathogenic nsSNP in some populations. This is because (i) *K10*-p.E443K is evolutionally highly conserved and the hydrophobic interaction with *K1*-p.L475 will disappear as the substituted amino acid “K” is hydrophilic; (ii) The first rotamer of p.E443K still has four clashes with p.K439 of *K10* after the energy minimization; (iii) the missense substitution p.E443K occurs on the ‘a’ position of the heptad repeat which is used to form the hydrophobic core which is very important for the formation of the *K1/K10* coiled-coil heterodimer. Meanwhile, for other *K1/K10* SNPs, the substituted amino acids are devoid of interchain interactions, so these missense mutations (SNPs) may not have any dominant negative effect on keratin intermediate filament polymerization. Hence, unlike disease causing pathogenic mutation, SNPs do not show any clinical manifestation.

**Table:** *In silico* prediction results of stability and pathogenicity of all variants

	AA change	DUET	Panther	SNPs3D
SNPs of Keratin 1	p.R403C	Destabilizing	-6.81647	-3.41
	p.D428E	Destabilizing	-2.03046	1.77
	p.Q431H	Destabilizing	-5.36226	-1.2
	p.R432C	Destabilizing	-6.35305	-0.68
	p.R432H	Destabilizing	-4.01658	0.01
	p.N435I	Stabilizing	-1.92249	1.1
	p.D439G	Destabilizing	-5.25381	-1.1
	p.A450G	Destabilizing	-5.1652	-2.38
	p.D457Y	Destabilizing	-6.3458	-2.2
	p.R463H	Destabilizing	-5.24828	0.3
	p.D464N	Destabilizing	-3.58309	-1.46
Keratin 1	p.L437P	Destabilizing	-6.01105	-2.76
	p.E478K	Destabilizing	-4.96195	-2.13
	p.E478Q	Destabilizing	-5.5345	-1.44
	p.E478D	Destabilizing	-4.61335	-1.79
	p.I479F	Destabilizing	-7.92481	-2.13
	p.I479T	Highly Destabilizing	-7.86206	-2.47
	p.T481P	Destabilizing	-5.99113	-2.47
	p.Y482C	Destabilizing	-10.9208	-3.16
	p.L485P	Destabilizing	-7.10998	-3.16
	p.L486P	Destabilizing	-7.06217	-3.16
	p.L486R	Destabilizing	-6.91351	-3.16
SNPs of Keratin 10	p.Q380H	Destabilizing	-6.04084	-1.38
	p.R399H	Destabilizing	-4.26811	-1.04
	p.Q410E	Destabilizing	-4.15495	-0.01
	p.Q420E	Destabilizing	-2.17831	1.35
	p.Q420H	Destabilizing	-5.32046	1.35
	p.E443K	Destabilizing	-6.92534	-1.38
	p.R450C	Destabilizing	-4.03936	0.32
	p.S451G	Destabilizing	-2.74555	1.74
Keratin 10	p.K439E	Destabilizing	-5.67407	-0.55
	p.L442Q	Highly Destabilizing	-7.56636	-2.41
	p.E445K	Destabilizing	-6.92534	-1.38
	p.I446T	Highly Destabilizing	-6.49942	-1.73
	p.Q447P	Stabilizing	-3.6748	0.12
	p.Y449D	Highly Destabilizing	-10.5608	-2.76
	p.Y449C	Destabilizing	-10.9208	-2.41
	p.R450P	Destabilizing	-6.15927	-2.09
	p.L452P	Destabilizing	-7.53639	-2.76
	p.L453P	Destabilizing	-7.41949	-2.41

**Figure 5:**
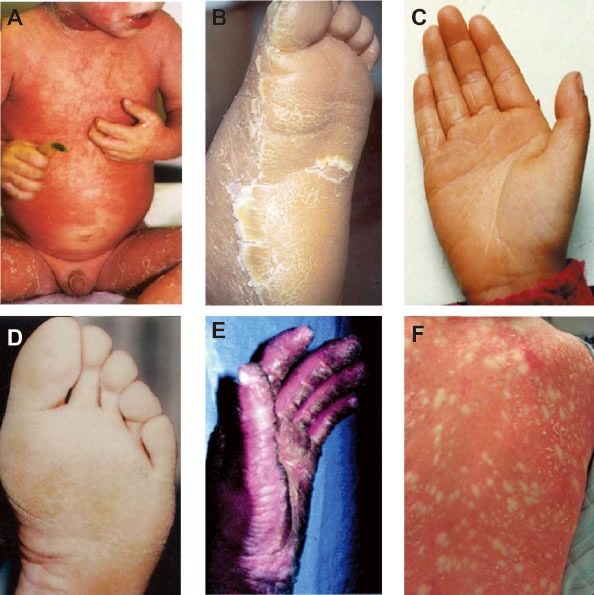
Clinical characteristics **A.** Clinical picture of BCIE from Virginia P. Sybert *et al.*, 1999©; Explosive gyrate erythema and peeling in patient. **B.** EHK from Paul E. Bowden *et al.*, 2003©; Hyperkeratosis and erythema in sole epidermis. **C.** PPK from K Xia *et al.*, 2009©; Hyperkeratotic plaques on palmar epidermis. **D.** CIEH from Virginia P. Sybert *et al.*, 1999©; Hyperkeratosis of soles. **E.** IHCM from Gabriele Richard *et al.*, 2001©; localized thickening or keratoderma can give the appearance of ridges or spikes on the palmar epidermis. **F.** CRIE from Richard P. Lifton *et al.*, 2010© slowly enlarging islands of normal skin surrounded by erythematous ichthyotic patches in a reticulated pattern. **A.**-**D.** are the genodermatoses caused by the missense mutations on 2B domain of *K1/K10* heterodimeric complex.

## DISCUSSION

In this study, we analyzed the molecular effects of missense mutations on keratin intermediate filament (KIF) polymerization. Our results clearly showed that missense mutations exert dominant negative effects on the *K1/K10* protein structure by altering inter-chain interactions and inter-atomic steric clashes. These local effects on structure propagate to affect stability of the *K1/K10* heterodimer, which in turn disrupts formation of the 10 nm keratin pancytoplasmic network.

**Figure 6:**
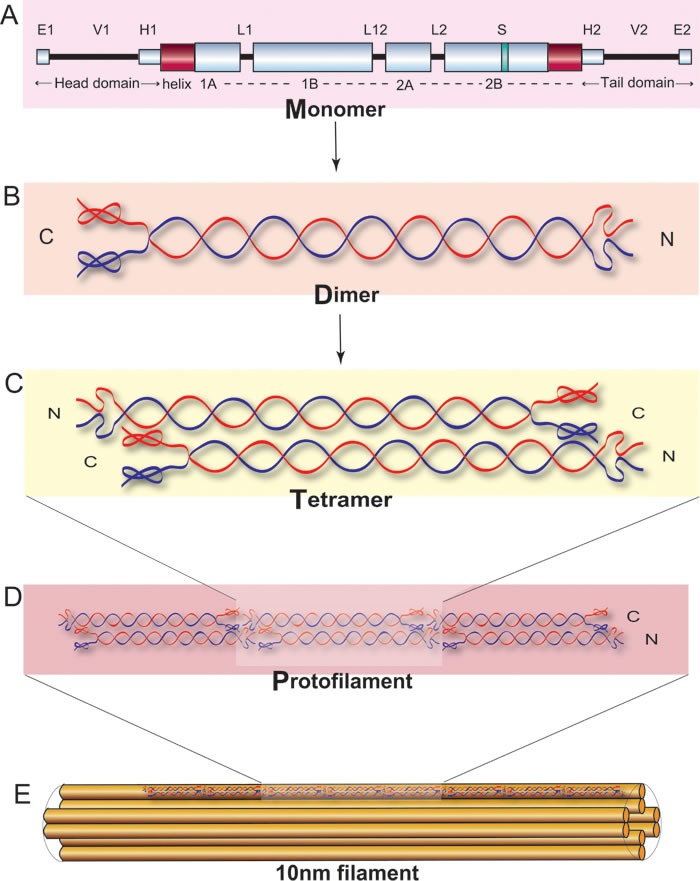
Schematic representation of keratin polymerized to the coiled-coil structure adapted from Figure 3 of Banerjee *et al.*, 2014©[4].

### Effect of environmental factors on severity of symptoms

One of the most significant features of genodermatoses associated with *K1/K10* mutations is that often the same mutation leads to variable range of phenotypes in a population specific manner. Notably, the same amino acid substitution in a specific position on *K1* causes different phenotypes in patients belonging to different geographical regions. One possible explanation for this phenomenon is the temperature and relative humidity sensitivity of symptoms. It has been reported that a pathogenic mutation (p.S233L) in 1B domain of *K1* causes a moderate form of EPPK in patients from Netherlands [12] whereas the same mutation in patients from Germany causes a severe form of NEPPK [13]. The difference in clinical severity can be explained by the local climate: Netherland has a moderate maritime climate, with cool summers and mild winters while Germany has a temperate seasonal climate dominated by humid westerly winds with a mild winter and warmer summer. The mean annual temperature of Germany is much higher than that of Netherlands and in most cases, severity of symptoms in genodermatoses are directly correlated with high ambient temperature. In order to understand the interactions between environmental and genetic factors on disease severity, further prolonged study is required.

### Evolutionarily conservation of amino acids: a significant factor

Among the amino acids in the 2B domain of *K1* and *K10*, those which are evolutionarily most conserved (across different species), cause the worst clinical symptoms upon missense mutation [*K1*: p.L486, *K10*: p.L453] (see Figure 2(vi)). The amino acids which are moderately evolutionarily conserved across different species cause moderate or mild phenotypes upon missense mutation [*K1*: p.L437]. Lastly, the amino acids which are not at all evolutionarily conserved, show no phenotype upon missense mutation, and are mostly referred as SNPs [*K1*: p.D457].

### Pathogenicity comparison between *K1/K5* and *K10/K14* over conserved amino acids

Keratin is a large family of genes belonging to either type I or type II keratin. *K1* is type II while *K10* is type I. Multiple sequence alignment across all type I and type II keratin genes showed that type I and type II keratin genes have a high degree of sequence similarity. As *K1* and *K5* both belong to the type II class, we found that mutation in the same conserved amino acids in *K1* or in *K5* always results in genodermatoses. In addition, as *K10* and *K14* both belong to the type II class, we found a similar correspondence for conserved amino acid mutations (see the comparison results in Suppl. Table 2).

### Significance of *TYRRLLEGE* at the helix termination motif

Genodermatoses associated with *K1* and *K10* mutations at the helix termination motif (HTM, the end of C-terminal tail domain) usually lead to the severe phenotype. Multiple sequence alignment across type I and type II keratin proteins showed that the HTM is evolutionarily well conserved in all type I type II keratin proteins. Several mutations causing severe phenotype have been reported in the helix termination motif for both the *K1* (p.L486R) and *K10* (p.L452P, p.L453P) polypeptides. A possible structural explanation for the importance of the HTM is its role in polymerization of the keratin protein filament. The *TYRRLLEGE* is an important region of molecular overlap, and controls both filament width and interaction between *K1/K10* heterodimers during filament elongation.

Since the helix boundary motifs at the beginning and the end of the rod domain are critical for normal filament formation, mutations located here cause the most severe form of genodermatoses.

### Mutations in the tail domain of *K10* always cause Erythroderma, ichthyosiform, congenital reticular (CRIE)

A very interesting feature of genodermatoses associated with *K10* mutations is that mutations in the tail domain of *K10* always lead to the severe phenotype termed Erythroderma, Ichthyosiform, Congenital Reticular (CRIE). CRIE is an extremely rare autosomal dominant disease with only seven patients clinically described in detail with four reported mutations in the tail domain of *K10* [14,15]. We postulated the following possible causes for the severe phenotype.

(i) *K10* is highly expressed in the suprabasal layers of the epidermis and forms heterodimers with *K1* [16,17], which then assemble to form 10 nm intermediate filaments. CRIE is caused by dominant mutations in *K10*, producing an arginine-rich C-terminal peptide that confers mislocalization of the protein to the nucleolus. This mislocalization provides a mechanism for disruption of the keratin filament network, which in turn contributes to loss of barrier function.

(ii) *K10* has previously been proposed to play a role in cell cycle regulation [18]. One possible causal mechanism for the mutations causing ichthyosis with confetti (IWC) is that they disrupt general cellular functions such as ribosome biogenesis, protein synthesis, DNA repair and replication. The nuclear localization of mutant *K10* is likely attributable to RNA binding owing to the extremely arginine-rich frameshift peptide and the high concentration of ribosomal RNA in the ribosome assembly factory; virtually all RNA binding proteins have arginine rich motifs that interact with the phosphate backbone of RNA, and the set of arginine-rich sequences capable of binding to RNA are diverse [19,20]. Similarly, arginine-rich motifs also contribute to nuclear localization [21].

(iii) Somatic reversion/mosaicism: CRIE is perhaps most remarkable for its exceptionally high frequency of spontaneous reversion. The mechanism – mitotic recombination - represents the complement of a mechanism for producing somatic homozygosity for tumor suppressor mutations [18]. These above mentioned possible causes can explain the occurrence of severe phenotype.

**Figure 7:**
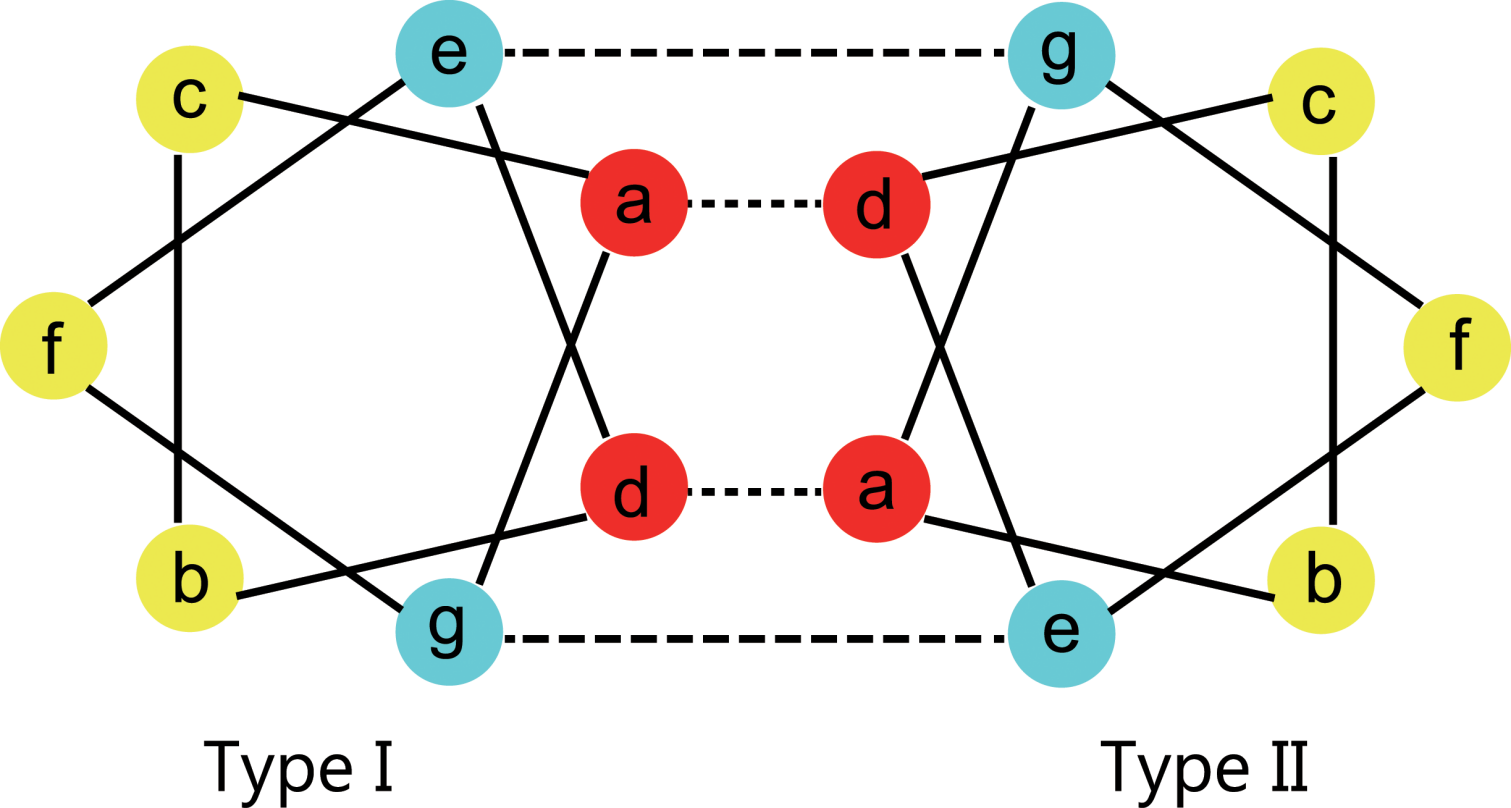
Heptad structure of the rod domain Schematic presentation of a transverse cut through the heptad repeats of the 2B domain for *K1* and *K10*, showing hydrophobic interactions between positions ‘a’ and ‘d’ (dashed lines) and ionic hydrogen interactions between positions ‘e’ and ‘g’ (dotted lines). As the two strands (*K1* and *K10* monomers) coil around each other, the positions ‘a’ and ‘d’ internalize and stabilize the structure, while positions ‘b, c, e, f’, and ‘g’ are exposed on the surface of the *K1/K10* heterodimer. Residues at positions ‘e’ and ‘g’ stabilize dimer formation through ionic and hydrogen bonds.

## MATERIALS AND METHODS

### *K1/K10* associated genodermatosis and its classification

*K1/K10* is associated with several types of genodermatosis with extreme phenotypic diversity. *K1/K10* associated genodermatoses most commonly display palmoplantarkeratoderma (hyperkeratosis of the palm and sole epidermis) and follicular hyperkeratotic papules with or without erythema as symptoms (Figure 5). However, due to the diversity of clinical symptoms, histological examination is the primary way of obtaining an accurate diagnosis. Many of the *K1/K10* genodermatosis arise from mutations on the 2B domain (examples shown in Figure 5A-D). For the remainder of this article, we will focus on missense mutations on the 2B domain linked to three diseases which we define below: BCIE/EHK, NEPPK and CIEH.

(1) Bullous erythroderma ichthyosiformis congenita of Brocq, bullous congenital ichthyosiform erythroderma, epidermolytic hyperkeratosis (BCIE/EHK) [MIM#113800]

BCIE/EHK is characterized by redness (erythroderma), blistering (bullous) and hypertrophy (ichthyosis-like) of the skin. Blistering lesions are seen at birth or soon after. It can be fatal in newborn infants because of secondary infections. Blistering subsides with age but patients develop progressively thickened hyperkeratotic skin, especially at flexures. In severe cases, this hyperkeratosis can be crippling. It has been shown that the nature of the keratin mutations is correlated with disease severity in BCIE, as *K1* associated BCIE-EHK gives rise to severe palmoplantar hyperkeratosis while *K10* associated BCIE/EHK lacks palmoplantar involvement.

(2) Non-Eepidermolyticpalmoplantarkeratoderma (NEPPK) [MIM#600962] [MIM#144200]

Non-epidermolytic palmoplantar keratoderma (NEPPK) is characterized by focal palmoplantar keratoderma with oral, genital, and follicular lesions. The Unna-Thost form of palmoplantar keratoderma is clinically identical to Vorner disease but can be distinguished histologically by the absence of epidermolysis.

(3) Cyclic ichthyosis with epidermolytic hyperkeratosis (CIEH) [MIM#607602]

CIEH affected individuals show palmoplantar hypokeratosis combined with periodic flares of developing erythematous scaly round patches which can expand and cover large areas of the skin, lasting for a few weeks to several months before subsiding and disappearing, leaving only the hyperkeratosis.

(4) Ichthyosishystrix, Curth-Mackin type (IHCM) [MIM#146590]

This kind of ichthyosishystrix is a disorder in which localized thickening or keratoderma can give the appearance of ridges or spikes on the skin surface.

(5) Erythroderma, Ichthyosiform, Congenital Reticular (CRIE) [MIM#609165]

CRIE is a rare skin condition characterized by slowly enlarging islands of normal skin surrounded by erythematous ichthyosis patches in a reticulated pattern.

### Protein structure of *K1/K10*

Keratins are the largest family of fibrous structural proteins and constitute the intermediate filament cytoskeleton in the cytoplasm of all epithelial cells. There are at least 54 known keratin genes expressed in the human genome that have an important structural role in the epidermis and appendages [22]. Keratin intermediate filament (KIF) proteins are composed of a central α-helical rod domain, flanked by a non-helical N-terminal head and a C-terminal tail domain (see Figure 6). The α-helical rod domain consists of four helices 1A, 1B, 2A and 2B which are interlinked by three short non-helical linker segments, linkers 1, 12 and 2 respectively. The *K1* and *K10* monomer together form a coiled-coil heterodimer complex, which in turn intertwine in an anti-parallel fashion to form a tetramer followed by the subsequent formation of a protofilament. Multiple protofilaments intertwine together to form a coiled-coil 10 nm keratin filament which finally helps in creating the pan-cytoplasmic network in basal keratinocytes of the epidermis.

The amino acid sequences of the α-helical rod domain contain a seven residue repeat, referred as heptad repeats (abcdefg)_n_ (see Figure 7). Heptad repeats include two hydrophobic amino acids (‘a’ and ‘d’), three polar amino acids (‘b’, ‘c’ and ‘f’) and two charged amino acids (‘e’ and ‘g’). As the two strands (*K1* and *K10* monomers) coil around each other, the positions ‘a’ and ‘d’ internalize and stabilize the structure, while positions ‘b, c, e, f’, and ‘g’ are exposed on the surface of the *K1/K10* heterodimer. Residues at positions ‘e’ and ‘g’ stabilize dimer formation through ionic and hydrogen bonds. Therefore, mutations to amino acids located at the ‘a’ and ‘d’ positions of the heptad repeats disrupts filament assembly through the hydrophobic core. Mutations to amino acids at the ‘g’ and ‘e’ positions disrupt tetramer formation. Mutations to amino acids situated in the ‘b’, ‘c’ and ‘f’ positions of the heptad repeat disrupt tetramer and higher level interactions.

The 2B domain is one of the most typical mutation hotspots and harbors most of the pathogenic mutations (see Table 3). Thus, we here focus our study within the 2B domain, i.e. *K1* (amino acids: 373–493) and *K10* (amino acids: 340–460). We integrate and investigate 21 missense mutations (*K1*: 11, *K10*: 10) and 19 SNPs (*K1*: 11, *K10*: 8) taken from the literature and online public databases such as HGMD and interfile (Details about these mutations are given in Table 3).

**Table:** Overview of all variants in *K1* and *K10* causing BCIE/EHK, NEPPK and CIEH

	Mutation		Name of disease	Symptom level	Reference
	cDNA	AA			
***K1***	c.1310T>C	p.L437P	NEPPK	Mild	[Liu XP et al., 2009]
	c.1432G>A	p.E478K	BCIE/EHK	Moderate	[XunXKet al., 2002]
	c.1432G>C	p.E478Q	CIEH	Moderate	[ArinMJet al., 2011]
	c.1434G>T	p.E478D	BCIE/EHK	Mild	[Yang JM Met al., 1999; Tsubota A et al. 2008],
	c.1435A>T	p.I479F	BCIE/EHK	Moderate	[Sybert VP et al., 1999; Michael EJ et al, 1999]
	c.1436T>C	p.I479T	BCIE/EHK/CIEH	Moderate	[Sybert VP et al, 1999; ArinMJ et al, 2000; Terron-Kwiatkowski A et al 2004;Zeng YP et al, 2012]
	c.1441A>C	p.T481P	BCIE/EHK	Moderate	[Muramatsu S et al., 2005]
	c.1445A>G	p.Yr482C	BCIE/EHK	Moderate	[SyderAJet al., 1994]
	c.1454T>C	p.L485P	CIEH	Moderate	[ArinMJet al., 2011]
	c.1457T>C	p.L486P	BCIE/EHK/CIEH	Moderate	[Lee DY et al., 2002; ArinMJ et al, 2011]
	c.1457T>G	p.L486R	BCIE/EHK	Severe	[Osawa Ret al., 2011]

	c.1207C>T	p.R403C			dbSNP: http://www.ncbi.nlm.nih.gov/projects/SNP/snp_ref.cgi?geneId=3848
	c.1284T>G	p.D428E			
	c.1293G>C	p.Q431H			
	c.1294C>T	p.R432C			
	c.1295G>A	p.R432H			
	c.1304A>T	p.N435I			
	c.1316A>G	p.D439G			
	c.1349C>G	p.A450G			
	c.1369G>T	p.D457Y			
	c.1388G>A	p.R463H			
	c.1390G>A	p.D464N			
	Mutation		Name of disease	Symptom level	Reference
***K10***	c.1315A>G	p.K439E	BCIE/EHK	Mild	[Syderetal., 1994]
	c.1325T>A	p.L442Q	BCIE/EHK	Moderate	[Chipev CC etal., 1994]
	c.1333G>A	p.E445K	BCIE/EHK	Severe	[Betlloch Ietal., 2009]
	c.1337T>C	p.I446T	CIEH	Severe	[Suga Y etal., 1998]
	c.1340A>C	p.Q447P	BCIE/EHK	Mild	[Shethetal., 2007]
	c.1345T>G	p.Y449D	BCIE/EHK	Mild	[Makino Tetal., 2012]
	c.1346A>G	p.Y449C	CIEH	Mild	[ArinMJetal., 2011]
	c.1349G>C	p.R450P	BCIE/EHK	Unknown	[Kiritsi Detal., 2013]
	c.1355T>C	p.L452P	BCIE/EHK	Severe	[McLean WHetal., 1999]
	c.1358T>C	p.L453P	BCIE/EHK	Severe	[Virtanen Metal., 2001; Virtanen M etal., 2003]

	c.1140G>C	p.Q380H			dbSNP:http://www.ncbi.nlm.nih.gov/projects/SNP/snp_ref.cgi?geneId=3858
	c.1196G>A	p.R399H			
	c.1228C>G	p.Q410E			
	c.1258C>G	p.Q420E			
	c.1260G>C	p.Q420H			
	c.1327G>A	p.E443K			
	c.1348C>T	p.R450C			
	c.1351A>G	p.S451G			

### Model building and validation

There are no publically available crystal structures for *K1/K10* in the protein data bank (PDB). We thus approximate the *K1/K10* 3D structures by homology modeling. This is a valid approach as keratin belongs to a very well-known family of intermediate filament structural proteins, which share similar sequence and structure. By running pair-wise sequence similarity searching using NCBI-BLAST against PDB entries under default parameters, we found that *K5/K14* (PDB ID: 3TNU, corresponding only to the 2B domain), had 76% of sequence identity between *K1/K5* and 64% between *K10/K14*. Construction of the initial structure of the *K1/K10* heterodimeric coiled-coil complex involved two steps. Firstly, HOMCOS [23], a user-friendly web server was used to obtain the respective monomer structures of *K1* and *K10* according to the template of 3TNU. Second, we applied the MODELLER scripts in order to combine the two monomers. These two steps combine the two monomers generated by HOMCOS into a heterodimeric coiled-coil structure [24].

The hypothetical protein models were validated by two computational tools: PROCHECK [25] and VADAR [26]. PROCHECK examines various parameters such as the stereochemistry, H-bonds, the region of occupancy in the Ramachandran plot, van der Waals contacts, buried charged residues, and packing defects. VADAR version 1.8 analyzes the properties of models by calculating their electrostatic potentials, volumes, accessible surface areas, and hydrogen-bonding interactions.

On the other hand, the accuracy of the resulting *K1/K10* homology model is evaluated by calculating the root mean square deviation (RMSD) between the atomic coordinates of the model with the experimental structure of the target protein (see Suppl. Table 3). The RMSD values were calculated for both backbones with a python script in PyMOL 1.6. An animation describing this superimposed model is also available at Oncotarget official website.

### *In silico* analyses

In order to analyze the effects of the missense mutations on the *K1/K10* coiled-coil structure, we use several bioinformatics programs DUET [27], SNPs3D [28] and PANTHER [29] to predict the effect of missense mutations on the basis of overall stability, pathogenicity and evolutionarily conservation (see Table 2). We use these programs as fundamental and preliminary analyses designed to investigate the molecular mechanism behind disease phenotype. In addition, we use the programs Ligplot [30], CMA [31], ESBRI [32] to analyze the inter-/intra-chain interactions (*e.g*., hydrogen bonds, hydrophobic–hydrophobic, aromatic–aromatic, and aromatic–polar interactions) and salt bridges. We use the resulting outputs to understand the importance of different interchain interactions as well as interface surfaces (Figure 2).

According to the result of multiple sequence alignment, all the amino acids of the 2B domain of *K1* and *K10* proteins are classified into three categories. These categories shown in Figure 2 are: ‘.’ denoting evolutionarily conserved, ‘:’ denoting more conserved and finally ‘*’ denoting most conserved. Most of the genodermatoses associated mutations of 2B domain of *K1/K10* belong to the class “evolutionarily most conserved” with only two exceptions: p.L437P of *K1* (not conserved) and p.R450P (evolutionarily more conserved). The amino acids belong to “evolutionarily most conserved (*)”, cannot be replaced by any other amino acids because mutations of these amino acids were harmful to protein's function. Amino acids belong to “evolutionarily more conserved (:)” or “evolutionarily conserved (.)” can be replaced by other amino acids with similar physico-chemical properties. Amino acids that are not evolutionarily conserved at all can be replaced by any other amino acids because mutations of these amino acids were harmless to protein's function.

Most of the SNPs in the 2B domain of *K1/K10* are not evolutionarily conserved. Only five SNPs are considered conserved (*K1*: p.R403C, p.D457Y, p.D464N; *K10*: p.R399H, p.E443K). Hence, evolutionary conservation allows us to quickly distinguish pathogenic SNPs (Figure 2).

In addition, we used the UCSF Chimera program [33] to predict the atomic contacts (*i.e.* steric clashes) of mutated amino acids with their side chains. Note that clashes are unfavorable interactions where atoms are too close together while contacts denote all kinds of direct interactions (polar and nonpolar, favorable and unfavorable) including clashes. Mutated amino acids with different rotamers lead to different inter-atomic or steric clashes. Here, we select the five most probable rotamers and perform structure minimization using UCSF Chimera (AMBER ff12SB force field) to investigate if the bad clashes could be accommodated to reach a stable structure with minimized energy.

## CONCLUSIONS

This is the first comprehensive *in silico* analysis of all (21) genodermatoses associated missense mutations on the 2B domain of *K1* and *K10*. Our main contributions are (i) making a homology model of the 2B domain for the *K1/K10* heterodimeric coiled-coil complex; (ii) extracting and integrating all the missense mutations (on 2B domain of *K1/K10*) from existing literature and databases; and (iii) investigating the correlation between phenotype and structural effects based on physico-chemical and structural characteristics. We characterize all the potential structural changes which have a dominant negative effect on the stability of the *K1/K10* coiled-coil hetero-dimer complex. *In vivo* or *in vitro* experiments would be recommended for validating all predicted tendencies followed by developing therapeutics for accurate genetic counseling and prenatal diagnosis. This case also demonstrates the value of homology modeling for comprehensive analysis of pathogenic missense mutations. Overall, this work illustrates the potential of *in silico* analysis for further diagnostic capabilities and disease understanding in clinical genetics.

## SUPPLEMENTARY MATERIAL TABLES


