# Supplementary material for: In silico predicted structural and functional insights of all missense mutations on 2B domain of K1/K10 causing genodermatoses
Source: Oncotarget. 2016 Jul 13;7(33):52766–80. doi: 10.18632/oncotarget.10599 (PMC5288147; doi:10.18632/oncotarget.10599)
Supplement: Supplementary file 1 [file oncotarget-07-52766-s001.pdf]

# *In silico* predicted structural and functional insights of all missense mutations on 2B domain of *K1/K10* causing genodermatoses

## Supplementary Material

**Suppl. Table 1. The clash analyses of the mutations.**

| | |
|---|---|
| *K1*-p.E478D | R1 (60.9%): No Clash. |
| | R2 (15.3%): No Clash. |
| | R3 (12.7%): 5 contacts with p.Y482 of K1, but after 52 steps clash disappeared. |
| | R4 (3.1%): 5 contacts with p.Y482 of K1, but after 14 steps clash disappeared. |
| *K1*-p.E478K | R1 (22.1%): No Clash. |
| | R2 (19.7%): 13 contacts with p.Y482 of K1 and p.I446, p.R450 of K10, and until 100 steps clash cannot disappear. |
| | R3 (6.1%): 13 contacts with p.Y482 of K1 and p.I446, p.R450 of K10, but after 37 steps clash disappeared. |
| | R4 (5.0%): 14 contacts with p.Y482 of K1 and p.I446, p.R450 of K10, and until 100 steps clash cannot disappear. |
| *K1*-p.E478Q | R1 (20.8%): No Clash. |
| | R2 (14.6%): 5 contacts with p.Y482 of K1, and until 100 steps clash cannot disappear. |
| | R3 (11.8%): 6 contacts with p.Y482 of K1, and until 100 steps clash cannot disappear. |
| | R4 (9.4%): No Clash. |
| *K1*-p.I479T | R1 (87.6%): No clash |
| | R2 (11.6%): No clash |
| | R3 (0.8%): 1 contact with p.D476 of K1, but after 1 step clash disappeared. |
| *K1*-p.L486P | R1 (53.1%): 6 contacts with p.Y482, p.R483 of K1, and until 100 steps clash cannot disappear. |
| | R2 (46.9%): 5 contacts with p.Y482, p.R483 of K1, and at the 8th step clash disappeared. But the clash appeared again from the 9th step. |
| *K1*-p.L486R | R1 (14.2%): 15 contacts with p.Y449 of K10, but after 18 steps clash disappeared. |
| | R2 (10.6%): 8 contacts with p.Y449 of K10, but after 44 steps clash disappeared. |
| | R3 (5.9%): No Clash. |
| | R4 (5.9%): 2 contacts with p.Y449 of K10, but after 7 steps clash disappeared. |

**The clash analysis of top four damaged SNPs**

| | |
|---|---|
| *K1*-p.R403C | R1 (69.7%): No clash. |
| | R2 (23.3%): No clash. |
| | R3 (7.0%): 1 contact with p.E400 of K1, but after 1 step clash disappeared. |
| *K1*-p.D464N | R1 (55.3%): No clash. |
| | R2 (11.3%): 2 contacts with p.Y432 of K10, but after 5 steps clash disappeared |
| | R3 (9.6%): No clash. |
| | R4 (5.7%): 1 contacts with p.Y432 of K10, but after 3 steps clash disappeared |
| *K10*-p.R399H | R1 (38.6%): 3 contacts with p.Q403 of K10, and until 100 steps clash cannot disappear. |
| | R2 (19.2%): 4 contacts with p.Q403 of K10, and until 100 steps clash cannot disappear. |

| | |
|---|---|
| | R3 (14.0%): 3 contacts with p.E395 of K10, and until 100 steps clash cannot disappear. |
| | R4 (11.1%): 2 contacts with p.E395 of K10, and until 100 steps clash cannot disappear. |
| *K10*-p.E443K | R1 (22.1%): 4 contacts with p.K439 of K10, but after 16 steps clash disappeared. |
| | R2 (19.7%): No clash. |
| | R3 (6.1%): No clash. |
| | R4 (5.0%): No clash. |

#1: We only select top four rotamer of each point mutation to show in detail.

#2: R1 (22.9%) means the first rotamer whose probability is 22.9%.

#3: Every showing clash is after structure minimization.

**Suppl. Table 2. Pathogenicity comparison between *K1/K5* and *K10/K14* at evolutionarily well conserved positions.**

| *K1* Mutation | Disease | References | Same mutation in *K5* | Disease | Reference |
|---|---|---|---|---|---|
| p. L437P | NEPPK | Liu et al, 2009 | p.L425 | No EBS Reported | |
| p. E478D | BCIE/EHK | Yang et al, 1999/ Tsubota et al, 2008 | p.E466D | EBS-K | Oh et al, 2007, Kang et al, 2010 |
| p.E478K | BCIE/EHK | Sun et al, 2002 | | | |
| p.E478Q | CIEH | Arin et al, 2011 | | | |
| p.I479F | BCIE/EHK | Sybert et al, 1999, Michael et al, 1999 | p.I467L | EBS-WC | Rugg et al, 2007 |
| p.I479T | BCIE/EHK, EPPK, CIEH | Sybert et al, 1999, Arin et al, 2000, Terron-Kwiatkowski et al, 2004, Arin et al, 2011,Zeng et al, 2012 | p.I467M | EBS-K | Pfendner et al, 2005; Bolling et al, 2011 |
| | | | p.I467T | EBS-DM | Irvine et al, 1997; Arin et al, 2010 |
| p.T481P | BCIE/EHK | Muramatsu et al, 2005 | p.T469P | EBS-DM | Müller et al, 2006 |
| p.Y482C | BCIE/EHK | Syder et al, 1994 | p.Y470H | EBS-K | Minakawa et al, 2013 |
| p.L485P | CIEH | Arin et al, 2011 | p.L473 | No EBS Reported | |
| p.L486P | BCIE/EHK, CIEH | Lee et al, 2002, Arin et al, 2011 | p.L474 | No EBS Reported | |
| p.L486R | BCIE/EHK | Osawa et al, 2011 | | | |

| *K10* Mutation | Disease | References | Same mutation in *K14* | Disease | Reference |
|---|---|---|---|---|---|
| p. K439E | BCIE/EHK | Syder et al, 1994 | p.K405 | No EBS Reported | |
| p. L442Q | BCIE/EHK | Chipev et al, 1994 | p.L408M | EBS-WC | Schuilenga-Hut et al, 2003 |
| p. E445G | BCIE/EHK | Betlloch et al, 2009 | p.E411K | EBS-DM/EBS-K | Glász-Bóna et al, 2009; Kaneko et al, 2011 |
| p. I446T | CIEH | Suga et al, 1998 | p.I412F | EBS-WC | Bolling et al, 2011; Bchetnia et al, 2012 |
| | | | p.I412N | EBS-DM | Glász-Bóna et al, 2009 |
| p. Q447P | Not conserved | | | | |
| p. Y449D | BCIE/EHK | Makino et al, 2012 | p.Y415H | EBS-K/EBS-DM | Hut et al, 2000;Rugg et al, 2000; Abu Sa'd et al, 2006; Kang et al, 2010; Jeřábková et al, 2009; Arin et al, 2010 |
| p.Y449C | CIEH | Arin et al, 2011 | p.Y415C | EBS-WC/EBS-K | Ciubotaru et al, 2003; Minakawa et al, 2013 |
| p. R450P | BCIE/EHK | Kiritsi et al, 2013 | p.R416P | EBS-DM | Wood et al, 2003 |
| p. L452P | BCIE/EHK | McLean et al, 1999 | p.L418V | EBS-K | Rugg et al, 2007 |
| p. L453P | BCIE/EHK | Virtanen et al, 2001, Virtanen et al, 2003. | p.L419Q | EBS-DM | Hut et al, 2000; Schuilenga-Hut et al, 2003 |

**Suppl. Table 3. Comparison between *K1/K10* homology model and *K5/K14* crystal structure.**

| Mode of comparison | R.M.S. deviation (Å) | Number of atoms |
|---|---|---|
| **All atoms** | 1.646 | 683 |
| **Backbone** | 1.140 | 336 |
